# Overexpression of the *FTSH* gene *GH_DO1G0225.1* enhances resistance against heat stress in cotton (*Gossypium hirsutum* L.)

**DOI:** 10.3389/fgene.2026.1784851

**Published:** 2026-05-15

**Authors:** Shahid Iqbal, Abdul Hafeez, Saadia Shehzad, Saima Bilal, Muhammad Imran, Muhammad Saleem Chang, M. Nasir Khan, Ghulam Rasool, Liu Fang, Muhammad Mubashar Zafar, Abdul Razzaq

**Affiliations:** 1 The Institute of Molecular Biology and Biotechnology, The University of Lahore, Lahore, Pakistan; 2 Department of Agronomy, Sindh Agriculture University Campus Umerkot, Sindh, Pakistan; 3 Department of Botany, University of Education, Lahore, Pakistan; 4 Department of Biological Sciences, Virtual University of Pakistan, Lahore, Pakistan; 5 Renewable Energy and Environmental Technology Center, University of Tabuk, Tabuk, Saudi Arabia; 6 The Institute of Cotton Research, Chinese Academy of Agricultural Sciences, Anyang, Henan, China; 7 Department of Plant Breeding and Genetics, University of Agriculture Faisalabad, Faisalabad, Pakistan; 8 International Center for Interdisciplinary Research in Sciences, The University of Lahore, Lahore, Pakistan

**Keywords:** climate change, gene transformation, gossypium, heat stress, transgenic cotton

## Abstract

**Introduction:**

Cotton is widely known as “white gold” due to its significant contribution to the global agricultural economy. Among the *Gossypium* species, the allotetraploid cottons *Gossypium hirsutum* and *Gossypium barbadense* are the most extensively cultivated due to their superior fiber yield and quality. However, rapid climatic fluctuations have intensified abiotic stresses, posing serious challenges to cotton production. Of these stresses, elevated temperature has emerged as one of the most damaging factors affecting cotton growth and productivity.

**Methods:**

In the current research, a genome-wide investigation of the filamentation temperature-sensitive H (*FTSH*) gene family was conducted to identify stress-responsive candidates. This analysis highlighted *GH_ DO1G0225.1* as a promising gene of interest. The candidate gene was confirmed through polymerase chain reaction (PCR) and found on a 1% agarose gel. Subsequently, *GH_DO1G0225.1* was successfully introduced in the cotton cultivar Ghauri genotype using an *Agrobacterium*-mediated transformation approach.

**Results:**

The observed transformation effectiveness as well as seed germination index were 1.89% and 71.42%, accordingly. Transcriptional profiling of the introduced gene was performed in the T0, T1, and T2 generations using quantitative real-time PCR, revealing consistently elevated expression levels. Compared with non-transgenic plants, transgenic cotton lines exhibited approximately 5.59-fold, 5.55-fold, and 5.45-fold increases in *GH_DO1G0225.1* expression in the T0, T1, and T2 generations, respectively.

**Discussion:**

Overall, the identification of *FTSH* family genes, followed by functional validation through genetic transformation, provides a valuable strategy for developing stress-resilient cotton cultivars and supports future efforts toward sustainable cotton production under adverse environmental conditions.

## 1 Introduction

Cotton is the domain’s most essential fiber and oil-producing crop (Patel et al., 2021; Salimath et al., 2021). Often referred to as “white gold,” it was farmed on over 33 million hectares internationally in 2019 (Tarazi et al., 2019). In Pakistan—an agriculture-based country—cotton is the second utmost main yield and plays a vital role in the national economy. It gives almost 0.8% to the nation’s overall GDP in addition to accounting for 4.5% of the cost extra to the agriculture region (Rehman et al., 2019). However, cotton production saw a notable decline of 17.5% in 2018–2019, dropping to 9.861 million rolls from 11.946 million rolls produced in 2017–2018. Out of all known cotton species, only four are commonly cultivated for field production. Among these, *Gossypium hirsutum* L and *Gossypium barbadense*, both members of the Malvaceae family, are the most widely grown around the world (Zafar et al., 2022). Together, they account for most of the worldwide cotton cultivation—*Upland cotton* makes up nearly 90% and *Gossypium barbadense* nearby 5% of the whole cotton acreage used for fiber making (Razzaq et al., 2022). Cotton fiber is primarily made of cellulose, accounting for approximately 88%–96.5% of its composition. The remaining non-cellulosic components—found mainly in the lumen or cuticle—include proteins (1.0%–1.9%), pectin (0.4%–1.2%), waxes (0.4%–1.2%), inorganic materials (0.7%–1.6%), and other substances (0.5%–8.0%) (Bowling et al., 2011).

Plants have to bear a change due to abiotic and biotic stresses throughout their growth. Drought, salinity, and heat are the most extensively studied abiotic stressors in the literature. Great heat stress has previously resulted in billions of dollars in crop damage globally because of increasing global warming (Inal et al., 2024).

The temperature-sensitive filamentation (*FtsH*) gene was first identified and named based on a temperature-sensitive mutant, ftsh1, which exhibited abnormal filament formation and was generated through chemical mutagenesis. The *FtsH* gene is part of the ATPases Associated with diverse cellular Activities (AAA+) protease family and contains three conserved structural domains: an N-terminal transmembrane domain, a C-terminal ATPase domain, and an M41 peptidase (protein hydrolase) domain (Janska, et al., 2013). *FtsH* orthologs were found to be nearly all cellular creatures, except a few archaeal species (Summer et al., 2006; Wagner et al., 2012; Giménez et al., 2015). For a while, the FtsH protein in *E. coli* was likewise referred to as HfIB, till it was determined that both names referred to the same gene (Herman et al., 1993). FtsH proteases are known for highlighting a wide variety of proteins—both membrane-bound and soluble—which play a role in regulating multiple cellular pathways (Kato and Sakamoto, 2018). The *FtsH* gene in plants was first discovered in spinach leaves (Lindahl et al., 1996) and later identified in other species such as tobacco (Pu et al., 2022), *Arabidopsis* (Liu et al., 2010), and rice (Wu et al., 2021). The proteins encoded by this gene are typically found in the membranes of chloroplasts and mitochondria. Most members have been found to perform a part in chloroplast improvement and maintaining cellular homeostasis, and are also involved in biological processes like vesicle formation (Li et al., 2022).

FtsH proteins are activated in response to various abiotic stresses and perform crucial part in helping plants cope with these conditions (Chen et al., 2018). For example, heat stress has been shown to trigger the appearance of *AtFtsH6* and *AtFtsH11* in *Arabidopsis* (Chen et al., 2018; Sedaghatmehr et al., 2016), *FtsH* genes in wheat (*Triticum aestivum*) (Rangan et al., 2020), and the *NEEDLE1* gene, a member of the FtsH family, in maize (*Zea mays*) (Liu et al., 2019). Similarly, drought and heat stress induce the appearance of *AtFtsH2* and *AtFtsH8* in *Arabidopsis* (Wang et al., 2014), as well as *FtsH8* in soybean (Das et al., 2016). Other FtsH proteins, including *AtFtsH1*, *AtFtsH5*, *AtFtsH6*, and *AtFtsH8*, are also upregulated by great brightness and temperature stress (Balfagón et al., 2019). Moreover, a lack of *AtFtsH11* in *Arabidopsis* results in increased understanding to heat (Chen et al., 2018), while overexpressing *TsFtsH8* from *Thellungiella salsuginea* improves icy stress tolerance (Liu et al., 2016). In tomatoes, *FtsH* expression is triggered in addition to heat shock as well as during certain growing phases (Sun et al., 2006). In *Arabidopsis*, the *FtsH6* gene is typically expressed at exact short stages below usual circumstances, as shown in the Arabidopsis eFP browser (http://bar.utoronto.ca/efp_arabidopsis; retrieved on 30 June 2025). However, underneath heat stress, *FtsH6*—specifically the plastid-localized metalloprotease encoded by *AT5G15250*—was the only one among 39 plastid protease genes to show a rapid increase in expression, according to heat-induced RNA-Seq data (Sedaghatmehr et al., 2016). In transgenic tomatoes, the *LeFtsH6* gene showed strong heat-induced GUS marking in leaves, roots, and flowers following heat treatment (Sun et al., 2006). Similarly, studies have reported that *FtsH6* expression is also upregulated by heat stress in other crops such as rapeseed (Yu et al., 2014), wheat (Xue et al., 2015), and sorghum (Johnson et al., 2014). Similarly, the *FTSH* gene has been reported in pepper (*Capsicum annum*) through *in silico* studies. However, experimental validation of its functional role under stress conditions has not yet been reported (Xiao et al., 2021). This consistent heat responsiveness suggests that *FtsH6* is a highly conserved nuclear gene across plant species and likely performs a vital part in the plant’s reaction to environmental stress (Kato and Sakamoto, 2018).

In plants, FTSH proteases are set through numerous genetic factors—*Arabidopsis thaliana*, for example, has 12 such genes (Adam et al., 2005; Wagner et al., 2012). The most well-studied of these is the hetero-complex located in the thylakoid membrane of chloroplasts. This complex consists of two subunit types: type A (FTSH1 and FTSH5) and type B (FTSH2 and FTSH8). While the proteins inside each category can functionally substitute for one another (i.e., are redundant), both types are necessary to form a stable and functional composite (Yu et al., 2004; Zaltsman et al., 2005; Adam et al., 2019; Sakamoto, 2006). This core compound—often referred to as FTSH1-2-5-8—along with the aforementioned counterparts in cyanobacteria and algae, plays a key role in repairing photosynthetic components that are damaged under high light conditions, particularly in cases of photoinhibition (Lindahl et al., 2000; Bailey et al., 2002; Kato et al., 2012; Komenda et al., 2012; Malnoë et al., 2014). It is likewise involved in responding to heat-stress-induced damage (Yoshioka et al., 2006; Yamamoto et al., 2008). In this work, a possible inconsistent manifested candidate gene (*GH_DO1G0225.1)* was recognized by the classification of the *FTSH* gene family (Iqbal et al., 2026). This candidate gene (*GH_DO1G0225.1*) needs to be confirmed by means of a practical experiment.

## 2 Materials and methods

### 2.1 Collection of materials

The seed of cotton variety Ghauri genotype was taken from the Department of Plant Breeding and Genetics, University of Agriculture Faisalabad, Faisalabad. The experiment was executed at FB Genetics Four Brothers Group, Lahore, and the Department of Plant Breeding and Genetics, University of Agriculture Faisalabad, Faisalabad.

### 2.2 Isolation of RNA and cDNA synthesis

Total RNA was isolated from samples of the cotton cultivar Ghauri genotype through the RNAprep Pure Plant Kit (Tiangen, Beijing, China). To eliminate any residual genomic DNA, the RNA preparations were treated with DNase I prior to downstream applications. RNA yield and purity were controlled spectrophotometrically with a NanoDrop 2000 instrument (Thermo Scientific, United States), and RNA reliability was additionally confirmed by separation on a 1% agarose gel. The resulting high-quality RNA was consequently reverse-transcribed into complementary DNA (cDNA) in a 20 μL reaction volume through the PrimeScript® RT Reagent Kit (Perfect Real Time; Takara Biotechnology Co., Ltd., Dalian, China). The synthesized cDNA was then diluted to a complete volume of 100 μL and stored for subsequent molecular analyses.

### 2.3 Detection of the *GH_DO1G0225.1* gene

Amplification of the *GH_DO1G0225.1* transcript was achieved from cDNA using gradient PCR across a temperature range of 55–65 °C. The thermal cycling program consisted of an early heat activation at 94 °C for 5 min, followed by 35 amplification cycles comprising denaturation at 94 °C for 30 s, primer annealing at 62.5 °C (selected based on a single and sharp band) for 35 s, and strand elongation at 72 °C for 30 s. A concluding extension step was carried out at 72 °C for an additional 5 min. PCR reactions were assembled in an ultimate volume of 25 μL, comprising 1 μL of cDNA template, 1 μL each of forward and reverse primers, 12.5 μL of Hi-Fi PCR master mix, and 9.5 μL of nuclease-free water. Amplification products were resolved on a 1% agarose gel prepared in freshly made 1× TAE buffer. Gene-specific amplification employed primers (Table 1).

**TABLE 1:** List of primer sequences.

Gene IDs	F’ primer	R’ primer	Product length (bp)
GH_D01G0225.1	CGC​CGC​GGC​TTT​CTG​AAA​AGC​C	CAC​GCT​CAC​CGG​GGT​CGG​CA	1902
GH_D01G0225.1	GTG​GCG​TAT​CAT​GAA​GTG​GG	AAA​TTC​ATC​GCC​GCT​CAT​GG	537

### 2.4 Cloning of the positive clones

For cloning, 4 μL of the purified PCR amplicon was mixed with 1 μL of a blunt-end TOPO cloning vector (TOPO TA Cloning Kit; Invitrogen, Cat. No. 45–0641) and incubated at 27 °C for 10 min in a thermal cycler to promote insert–vector joining. A portion of the ligation reaction (5 μL) was then gently combined with 50 μL of chemically competent *Escherichia coli* cells in a sterile microcentrifuge tube. The cell suspension was maintained on ice for 30 min before taking it for a short heat shock at 42 °C for 1 min, followed by rapid cooling on ice for approximately 2–3 min. Recovery was carried out by adding 450 μL of LB liquid medium (10 g/L tryptone, 5 g/L yeast extract, and 5 g/L NaCl) and incubating the cells at 37 °C with shaking at 200 rpm for 55 min. Transformed cells (25–100 μL) were spread onto LB agar plates accompanied by kanamycin (50 mg/L) and incubated at 37 °C for 12 h or overnight. Well-isolated colonies were subsequently selected and cultured in 5 mL of LB broth containing kanamycin (50 mg/L) at 37 °C with shaking. Plasmid DNA was finally separated from the overnight cultures through a commercial plasmid purification kit (Thermo Scientific, Cat. No. K0503).

### 2.5 Ligation and transformation of the gene into *A. tumefaciens*

The ligation and transformation of recombinant plasmid DNA obtained from confirmed clones to plant expression vector pCAMBIA2300 using restriction endonucleases *Bgl*II and *Nco*I, followed by a protocol described by Razzaq et al. (2021).

### 2.6 Confirmation of gene constructs in *A. tumefaciens* through PCR

Individual, well-separated colonies of *Agrobacterium tumefaciens* were picked from YEP solid medium and confirmed the presence of construct using optimum PCR conditions such as: preliminary denaturation step at 94 °C for 5 min, followed by repeated cycles of denaturation at 94 °C for 30 s, primer annealing at 62.5 °C for 35 s, and elongation at 72 °C for 30 s, with a final extension at 72 °C for 5 min. Each PCR reaction was assembled in a concluding volume of 25 μL, comprising 1 μL of template DNA, 1 μL of each primer, 12.5 μL of Hi-Fi PCR master mix, and 9.5 μL of nuclease-free water. Detailed information on primer sequences is provided in Table 1.

### 2.7 Transformation of the gene into cotton

**FIGURE 1:**
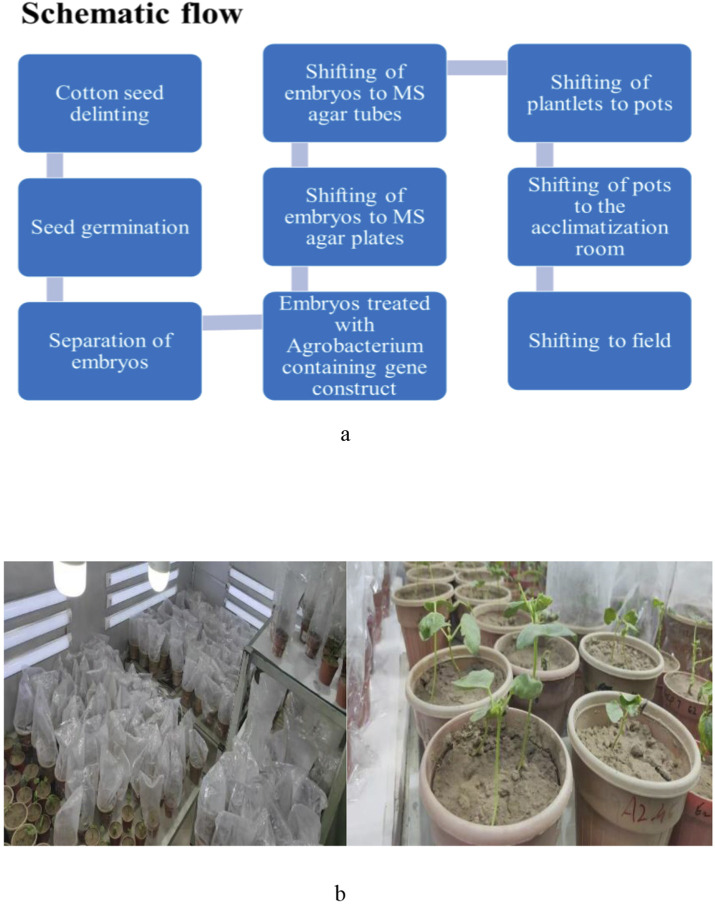
**(a)** Schematic flow of the transformation of the gene into the cotton variety Ghauri genotype. **(b)** The pictorial representation of the transformed plants.

Seeds of the cotton variety Ghauri genotype were delinted and disinfected before being maintained at 30 °C for 48 h to obtain uniformly germinated seedlings. These seedlings were taken for genetic modification employing a shoot-apex excision approach based on the method of Ulian et al. (1988). After mechanical injury, the embryos were inoculated with *Agrobacterium tumefaciens* strain LBA4404 following the method described by Razzaq et al. (2021). Measurements related to seed germination, transformation success, and the overall experimental scheme are concised in Supplementary Tables S2–S4 and Figure 1a, and a pictorial representation of the transformed is given in Figure 1b.

### 2.8 Detection of the gene in putative transgene cotton through PCR

Young leaf tissue from the putative transgenic cotton lines was used for molecular validation of the target gene (*GH_DO1G0225.1*) by PCR analysis. Amplification was performed according to the maker’s instructions provided with the Green Plant Direct PCR Master Mix kit (Thermo Scientific). Detection of the transgene was carried out using gene-specific primers designed to amplify a short fragment of the target sequence (Table 1).

### 2.9 RNA extraction and cDNA preparation

Gene-specific primer sets targeting *GH_DO1G0225.1* were made through Primer3 (version 4.0). Total RNA was isolated from leaf tissues of the putative transgenic cotton plants employing the Agilent RNA extraction kit (Agilent Technologies, Santa Clara, CA, United States). The application and clarity of the separated RNA were determined through a NanoDrop 2000 spectrophotometer (Thermo Scientific, United States) by calculating absorbance ratios at 260 and 280 nm. Following DNase treatment, purified RNA was used for first-strand complementary DNA (cDNA) synthesis with the PrimeScript® RT Reagent Kit (Perfect Real Time; Takara Biotechnology Co., Ltd., Dalian, China). The synthesized cDNA samples were subsequently preserved at −20 °C for downstream analyses.

### 2.10 Expression analysis of cotton transgene

Quantitative real-time PCR (qRT-PCR) analysis was executed to assess the expression level of the introduced gene in cotton plants by gene-specific primers that amplified a 542-bp fragment. Reactions were carried out in triplicate in accordance with the company’s directions for the Maxima® SYBR Green/ROX qPCR Master Mix (Thermo Scientific). Each 20 μL reaction mixture comprised of 1 μL each of forward and reverse primers (10 pmol), 5 μL of 2× Maxima® SYBR Green/ROX Master Mix, and 1 μL of cDNA template (50 ng/μL), with the remaining volume adjusted using nuclease-free water. Histidine was employed as the reference gene for data normalization, and all amplification assays were individually repeated three times.

*Histidine was selected as the reference gene for data normalization based on previous studies demonstrating its stable expression across various tissues, developmental stages, and stress conditions in cotton (Artico et al., 2010; Wang et al., 2019; Chen et al., 2020).

## 3 Results

### 3.1 Isolation of the *GH_DO1G0225.1* gene from cotton seeds

Mature seeds of the cotton cultivar Ghauri genotype were harvested and processed for the isolation of total messenger RNA, which was then reverse-transcribed to generate complementary DNA (cDNA). The concentration and integrity of the separated mRNA were verified through a NanoDrop 2000 spectrophotometer (Thermo Scientific, United States). Amplification of the target gene was carried out using gene-specific primers (Table 1). PCR amplification produced a clear fragment of 1902 bp, confirming successful amplification of the target sequence, as illustrated in Figure 1.

### 3.2 Cloning and plasmid isolation

**FIGURE 2:**
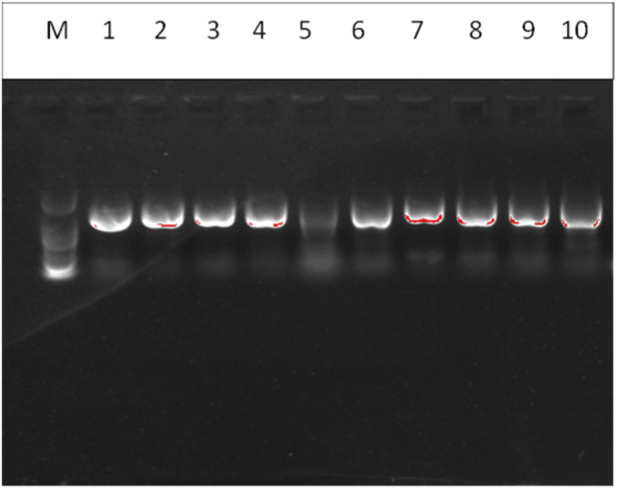
Gel electrophoresis of PCR products: 2; M shows molecular weight marker of 10kb, lanes 1–10 show isolated plasmids showing positive clones.

The PCR-purified amplicon was ligated into a Blunt Zero cloning vector and subsequently introduced into *Escherichia coli* DHα5 competent cells by transformation. Transformed colonies were randomly picked and screened by colony PCR to confirm the presence of the insert. Positive colonies were then propagated, and the resulting PCR-verified product was subjected to plasmid extraction, as illustrated in Figure 2.

### 3.3 Gene (*GH_DO1G0225.1*) integration into plant expression vector PCAMBIA2300

**FIGURE 3:**
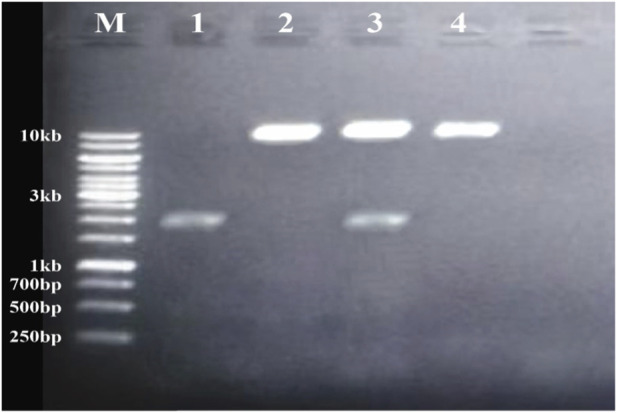
Gel electrophoresis of PCR products: M shows the molecular weight marker of 10kb, lane 1 shows the gene product size of 537 bp, lane 2 shows the expression vector, lane 3 shows the gene and expression vector without ligation, lane 4 shows ligation of gene and expression vector.

**FIGURE 4:**
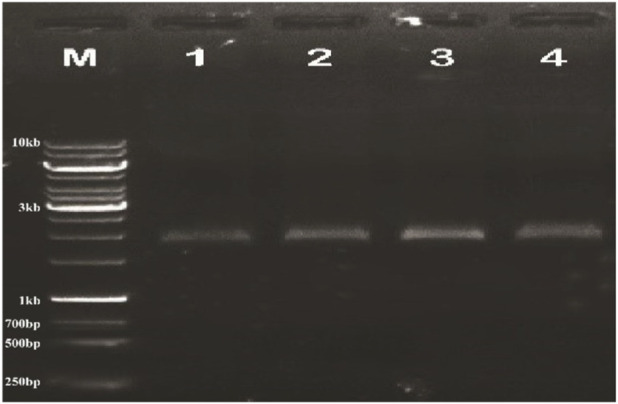
Gel electrophoresis of PCR products: M shows the molecular weight marker of 10kb, lanes 1-4 show gene product size of 1902 bp using full-length gene-specific primers.

**FIGURE 5:**
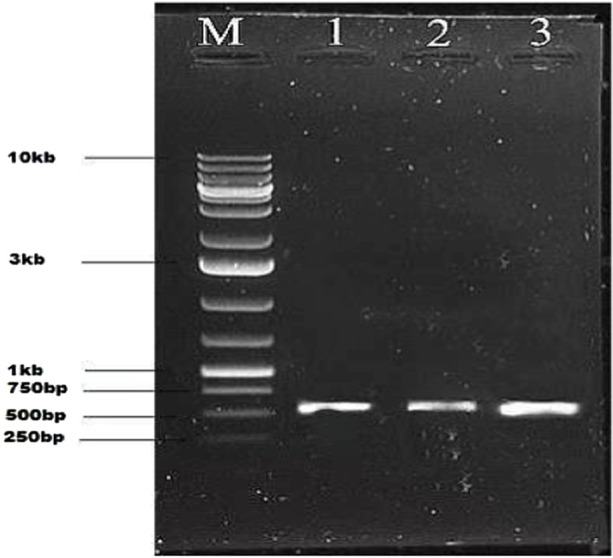
Gel electrophoresis of PCR products: M shows the molecular weight marker of 10kb, lanes 1-3 show gene product size of 537 bp using short-length gene-specific primers.

Plasmid DNA isolated from confirmed recombinant clones and the plant expression vector pCAMBIA2300 were separately digested with the restriction enzymes *Bgl*II and *Nco*I. The appropriate DNA fragments were excised from agarose gels, purified, and subsequently joined using the Fast Ligation Kit (Thermo Scientific; Cat. No. K1423). The resultant ligation mixture was introduced into *Escherichia coli* DHα5 competent cells. Successful assembly of the recombinant construct was verified by molecular analysis (Figure 3), after which the validated plasmid was transferred into *Agrobacterium tumefaciens* via a liquid nitrogen–mediated transformation procedure. Confirmation of the recombinant *Agrobacterium* strains was achieved by PCR using both full-length and truncated gene-specific primer sets. Amplification products of 1902 bp with the full-length primers and 537 bp with the short-fragment primers verified the successful incorporation of the target gene into *Agrobacterium*, as shown in Figures 4, 5.

### 3.4 Genetic transformation of cotton with *GH_DO1G0225.1*

**FIGURE 6:**
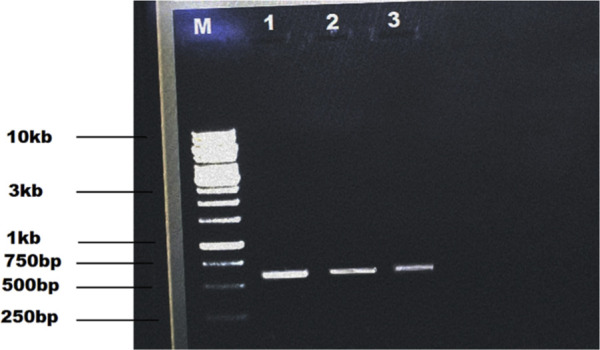
Gel electrophoresis of PCR products: M shows the molecular weight marker of 10kb, lanes 1-2 show the product size of 537 bp.

Seeds of the cotton cultivar Ghauri genotype were first delinted and disinfected, then maintained at 30 °C for 48 h to ensure uniform sprouting. The resulting seedlings were subjected to genetic modification using the shoot-apex incision technique to introduce the *GH_DO1G0225.1* gene. Following initial selection and confirmation steps, genomic DNA was isolated from leaf tissues of the putative transgenic plants. Integration of the target gene was evaluated by PCR using short amplicon–specific primers, which yielded a distinct 537 bp product, thereby verifying successful incorporation of *GH_DO1G0225.1* into the cotton genome, as illustrated in Figure 6.

### 3.5 Tracking expression of the gene *GH_DO1G0225.1* in putative transformants of cotton

**FIGURE 7:**
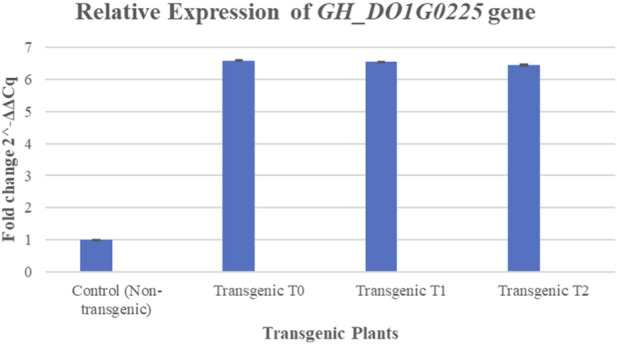
Comparative expression analysis of transgenic (T_0_, T_1_, and T_2_) and non-transgenic cotton plants.

Transcript accumulation of *GH_DO1G0225.1* was quantified in three independent transgenic cotton lines using quantitative real-time PCR (qRT-PCR) with gene-specific primers and the Maxima SYBR Green/ROX detection chemistry (Thermo Scientific, United States). All reactions were conducted in three technical replicates, and histidine served as the endogenous reference gene for data normalization. Expression analysis was performed across successive generations (T_0_, T_1_, and T_2_) of the putative transformants. Comparative analysis revealed generation-dependent variation in relative transcript abundance (Figure 7), with elevated expression levels of *GH_DO1G0225.1* observed in transgenic plants. The Y-axis shows relative fold expression; the X-axis shows transgenic plants, whereas the error bars show standard deviation. Specifically, transcript levels increased by approximately 5.59-fold in T_0_, 5.55-fold in T_1_, and 5.45-fold in T_2_ plants relative to non-transformed controls. Statistical analyses were performed using one-way analysis of variance (ANOVA) to compare expression levels among T_0_, T_1_, T_3,_ and non-transgenic control plants. A p-value <0.05 was considered statistically significant.

## 4 Discussion

Undoubtedly, cotton is considered the backbone of the economy of different countries such as the United States, Argentina, Brazil, China, India, Turkey, and Pakistan (Salimath et al., 2021). Pakistan is an agriculturally based country, and its economy is directly associated with cotton production. It contains almost 0.8% to the nation’s overall GDP and accounts for 4.5% of the cost additional to the agriculture area (Rehman et al., 2019).

The *FtsH* gene was first recognized and named based on a temperature-sensitive mutant, called ftsh1. It exhibited abnormal filament formation, which was generated through chemical mutagenesis. The *FtsH* gene is part of the AAA + protease family and contains three conserved structural domains: an N-terminal transmembrane domain, a C-terminal ATPase domain, and an M41 peptidase (protein hydrolase) domain (Janska, et al., 2013). FtsH orthologs were lately found in nearly every cellular being, excepting a few archaeal species (Summer et al., 2006; Wagner et al., 2012; Giménez et al., 2015). The FTSH gene family has been reported in prokaryotes and eukaryotes. It performs a key part in progress and improvement, and resistance to abiotic stress (Wu et al., 2021). The FTSH gene family has been recognized in *Arabidopsis thaliana*, rice, maize, and soybean, but it has not been reported in cotton.

FtsH proteins are activated in response to various abiotic stresses and have crucial parts in helping plants cope with these conditions (Chen et al., 2018). For example, heat stress has been shown to trigger the expression of *AtFtsH6* and *AtFtsH11* in *Arabidopsis* (Chen et al., 2018; Sedaghatmehr et al., 2016), *FtsH* genes in wheat (*Triticum aestivum*) (Rangan et al., 2020), and the *NEEDLE1* gene, a member of the FtsH family, in maize (*Zea mays*) (Liu et al., 2019). Currently, the cotton yield is facing various biotic and abiotic stresses to meet the consumption requirement. Due to climate change, among the abiotic stresses, heat stress has become a major concern worldwide. Therefore, the current study was designed to result in proposing a solution.

Due to the similarity of the FTSH protein with that in *A. thaliana*, the study was designed, and the *FTSH* gene family was characterized. It was found that the *FTSH* gene family has the potential to contribute to resistance against abiotic stress. Based on genome-wide research, a possible inconsistently demonstrated candidate gene (*GH_DO1G0225.1)* was acknowledged in a previous study (Iqbal et al., 2026). Here, the identified candidate has been validated through transformation into cotton. The Agrobacterium-mediated transformation method was employed to validate the gene in terms of transformation efficiency and differential expression measured by qRT-PCR.

In this Research, the abiotic stress-matched gene (*GH_DO1G0225.1)* was recognized via genomic dispersal and evolutionary research. The gene was converted into a cotton assortment, Ghuari genotype, using Agrobacterium-mediated transformation. This came into observation that the overexpression of the gene (*GH_DO1G0225.1)* is engaged in generating resistance in the Ghauri genotype to heat stress. This research was consistent with (Rangan et al., 2020). The transformation proficiency and germination index were also estimated, resulted into 1.89% and 71.42% separately. The fold expression of transgenic cotton came in between 5.59-, 5.55-, and 5.45-folds greater in T_0_, T_1_, and T_2_ plants of transmuted cotton, individually, as related to the non-transgenic cotton plants. These consequences were stabilized by means of the earlier performed overexpression of genes into cotton (Chen et al., 2018). Therefore, our figures powerfully help the impression that the genes of the *FTSH* gene family associated with biotic stress, having a conserved domain, demonstrate resistance against heat stress.

The stable overexpression of *GH_DO1G0225.1* across T_0_, T_1_, and T_2_ generations (5.59-, 5.55-, and 5.45-fold, respectively) confirms the heritable integration of the transgene into the cotton genome. The absence of significant expression variation among generations indicates consistent transcriptional activity without transgene silencing, a common challenge in plant transformation (Matzke and Matzke, 1998). This stable inheritance supports the utility of *GH_DO1G0225.1* for breeding programs. Although the present study focused on molecular characterization, the sustained expression levels suggest that *FTSH* family members may contribute to protein quality control mechanisms under stress, consistent with their established roles in protecting photosynthetic machinery during heat stress (Kato and Sakamoto, 2018). Future functional assays are warranted to correlate expression levels with physiological tolerance.

## 5 Conclusion

The current natural research considered a functionally essential gene (*GH_DO1G0225.1)* that is familiar to the FTSH gene family in cotton. The current research revealed the recessives of the *GH_DO1G0225.1* gene in cotton have led to enhanced resistance to the transgenic cotton selection Ghauri genotype in contrast to heat stress. The molecular study indicated that the expression of the gene in transgenic plants was greater than that of non-transgenic plants. These molecular findings suggest that *GH_DO1G0225.1* is a promising candidate for further functional validation; however, additional physiological and phenotypic assays (e.g., survival rate, membrane stability, photosynthetic performance, and oxidative stress markers) are required to conclusively establish its role in heat stress tolerance. In addition, the appearance of the transgene in T_0_, T_1_, and T_2_ generations was steady as well as constant in the host genome. The stable expression of the transgene across successive generations supports the potential utility of this gene in future breeding programs aimed at developing stress-resilient cotton cultivars. This dominance of the genes eventually led to the development of cotton production. The substantial increase in the overexpression of a gene may play an important part in textile production as well as in sustainable agricultural performance. Our research also gives important evidence for additional investigation of the molecular mechanism of the *FTSH* gene family in cotton.

## Data Availability

The original contributions presented in the study are included in the article/Supplementary Material, further inquiries can be directed to the corresponding author.
