# Supplementary material for: Overexpression of the FTSH gene GH_DO1G0225.1 enhances resistance against heat stress in cotton (Gossypium hirsutum L.)
Source: Front Genet. 2026 May 15;17:1784851. doi: 10.3389/fgene.2026.1784851 (PMC13218948; doi:10.3389/fgene.2026.1784851)
Supplement: Supplementary file 1 [file DataSheet1.pdf]

Supplementary Tables 2-4

Table 2: Numerical data for transformation experiments

Exp. No.	No. of embryos isolated	Agrobacterium treated embryos	Embryos on MS plates	Died	Selection tubes	Plantlets died	Plants transferred to pots	plants died in pots	Plants shifted to greenhouse
1	115	110	103	96	7	3	4	2	2
2	111	105	105	99	6	3	3	1	2
3	103	97	97	87	10	4	6	2	4
4	95	92	92	88	4	1	3	1	2
5	109	105	105	96	9	3	6	3	3
6	169	166	166	157	9	3	6	2	4
7	181	178	178	170	8	3	5	2	3
Total	883	846	846	793	53	20	33	13	20

Table 3: Germination index

No. of Petri plates	Total seeds	No. of germinated seeds	No. of ungerminated seeds	Germination index
1	35	25	10	71.42%

Table 4: Transformation efficiency

Agrobacterium treated embryos	Control plants	Plants shifted to greenhouse		transformation efficiency	
		Control plants	Experimental	Control plants	Experimental
846	35	15	16	42.85%	1.89%
